# Retraction: Conversion of carbon black recovered from waste tires into activated carbon *via* chemical/microwave methods for efficient removal of heavy metal ions from wastewater

**DOI:** 10.1039/d5ra90139a

**Published:** 2025-11-19

**Authors:** M. M. El-Maadawy, Amir A. Elzoghby, Ahmed M. Masoud, Alzahraa M. Eldeeb, Ahmed M. A. El Naggar, Mohamed H. Taha

**Affiliations:** a Nuclear Materials Authority PO Box 530, El Maddi Cairo Egypt amirelzoghby33@gmail.com chemmaso010@hotmail.com; b Chemistry Department, Faculty of Science, Mansoura University Mansoura Egypt; c Egyptian Petroleum Research Institute (EPRI) 1 Ahmed El-Zomor St., Nasr City Cairo Egypt

## Abstract

Retraction of ‘Conversion of carbon black recovered from waste tires into activated carbon *via* chemical/microwave methods for efficient removal of heavy metal ions from wastewater’ by M. M. El-Maadawy *et al.*, *RSC Adv.*, 2024, **14**, 6324–6338, https://doi.org/10.1039/D4RA00172A.

The Royal Society of Chemistry hereby wholly retracts this *RSC Advances* article due to concerns with the reliability of the data.

The EDX spectra in Fig. 3 show identical sections in C and D, and duplicated sections within the two spectra. The authors state they were only provided with the image file and therefore have not been able to provide the raw data for the published version but have provided repeated analysis. The author's response has been reviewed by an independent expert who has deemed it unsatisfactory.

Given the significance of these concerns, the Editor has lost confidence that the findings presented in this paper are reliable.

The authors were informed about the retraction of the article. Alzahraa M. Eldeeb, Ahmed M. A. El Naggar, Mohamed H. Taha, Ahmed M. Masoud, Amir A. Elzoghby and M. M. El-Maadawy have not agreed with the decision.

Signed: Laura Fisher, Executive Editor, *RSC Advances*

Date: 13^th^ November 2025

